# Pharmacokinetic Simulations of Intravitreal Biologicals: Aspects of Drug Delivery to the Posterior and Anterior Segments

**DOI:** 10.3390/pharmaceutics11010009

**Published:** 2018-12-30

**Authors:** Anna-Kaisa Rimpelä, Iiro Kiiski, Feng Deng, Heidi Kidron, Arto Urtti

**Affiliations:** 1 Drug Research Program, Faculty of Pharmacy, University of Helsinki, Viikinkaari 5 E, 00790 Helsinki, Finland; anna-kaisa.rimpela@helsinki.fi (A.-K.R.); iiro.kiiski@helsinki.fi (I.K.); feng.deng@helsinki.fi (F.D.); heidi.kidron@helsinki.fi (H.K.); 2 Laboratory of Biohybrid Technologies, Institute of Chemistry, St. Petersburg State University, Universitetskii pr. 26, Peterhoff, 198504 St. Petersburg, Russia; 3 School of Pharmacy, University of Eastern Finland, Yliopistonranta 1, 70211 Kuopio, Finland

**Keywords:** intravitreal injection, ocular drug delivery, bevacizumab, ranibizumab, aflibercept, controlled release

## Abstract

Biologicals are important ocular drugs that are be delivered using monthly and bimonthly intravitreal injections to treat retinal diseases, such as age-related macular degeneration. Long acting delivery systems are needed for prolongation of their dosing interval. Intravitreal biologicals are eliminated from the eye via the aqueous humor outflow. Thus, the anterior and posterior segments are exposed to the drug. We utilized a kinetic simulation model to estimate protein drug concentrations in the vitreous and aqueous humor after bolus injection and controlled release administration to the vitreous. The simulations predicted accurately the experimental levels of 5 biologicals in the vitreous and aqueous humor. The good match between the simulations and experimental data demonstrated almost complete anterior segment bioavailability, and major dose sparing with ocular controlled release systems. Overall, the model is a useful tool in the design of intraocular delivery of biologicals.

## 1. Introduction

Biologicals are widely used in the treatment of ocular diseases, such as age-related macular degeneration and uveitis [1,2]. In clinics, ranibizumab, aflibercept and bevacizumab are injected into the vitreous cavity to reach effective concentrations in the retina [3]. Antibodies are given intravenously for the treatment of uveitis and associated rheumatoid arthritis [4], but there is growing interest in the local uveitis treatment as exemplified by the development of an intravitreal fluocinolone acetonide implant [5]. During the last decade, intravitreal injections have become widely applied routine procedures in the clinics. The global number of intravitreal anti-VEGF injections has reached almost 20 million per year [6].

Intravitreal drugs are eliminated posteriorly across the blood retina barrier and anteriorly via aqueous humor outflow. The posterior route is relevant for the small molecular drugs that are able to permeate through the biological barriers (endothelia of the retinal capillaries, retinal pigment epithelium (RPE), barriers in the ciliary body) [7]. Vitreal half-lives of such small molecular drugs are less than 10 h, while half-lives of biologicals are in the range of one week [8]. Biologicals are mainly (at least 82% of the dose) eliminated via the anterior route, because they permeate poorly through the blood-ocular barriers [7,9,10,11]. Thus, the anterior chamber is exposed to the intravitreal protein; bioavailability in the aqueous humor being >82% [9,10].

Some diseases, for example uveitis and glaucoma, affect both anterior and posterior segments of the eye. In such cases, exposure of both parts to the injected drug may offer therapeutic benefits. Intravitreal delivery of biologicals has some advantages: a relatively long half-life (about one week), and high bioavailability in the vitreous (100%) and anterior chamber (>82%). Intracameral injection results in complete bioavailability in the aqueous humor, but the vitreal bioavailability of intracameral injections is poor, the half-life in the aqueous humor is about one hour, and unlike intravitreal injections, intracameral injections are not widely used in clinical practice like intravitreal injections. Topical application of biologicals is not feasible, because the bioavailability is practically zero in both anterior and posterior segments [7]. Sub-conjunctival injection may be feasible, but the bolus injections have short half-lives (hours) and the drug bioavailability is less than 10% to the anterior chamber, and about 0.1% to the retina [12,13]. Therefore, intravitreal delivery is an interesting delivery option for biologicals that have posterior and/or anterior targets.

Transport of biologicals from the vitreous to the anterior chamber has been quantitated in many experimental rabbit studies [14,15,16,17,18]. Furthermore, this has been modeled recently with physiologically based pharmacokinetic (PBPK) and finite element models [8,19]. Since PBPK and finite element models require specialized expertise in modeling, they are not accessible to most academic and industrial investigators in the field of ocular drug delivery. Therefore, we developed a simple model that is based on protein diffusion in the vitreous and its elimination via the anterior route.

## 2. Materials and Methods

### 2.1. Elimination of the Intravitreal Biological

**Figure 1:**
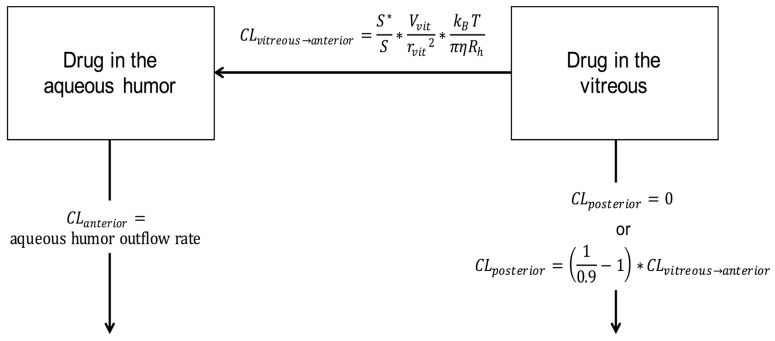
Schematic illustration of the simulation model.

The model structure is illustrated in Figure 1.

The model was built using the logic of Hutton-Smith et al. [8]. We assume that the protein drug is injected in the middle of rabbit vitreous. Thereafter, the protein will diffuse within the vitreous. The average diffusion time from the injection site in the rabbit vitreous to the border of vitreous and posterior chamber is estimated as:

(1) $$T_{diff}=\frac{r_{vit}{}^{2}}{6D}$$

where *D* is the diffusion coefficient of the protein drug in the vitreous and *r_vit_* is the diffusional pathlength in the vitreous. The diffusion coefficient depends on the molecular properties as defined in the Equations (2) and (3). For parameter values, see Table 1.

(2) $$D=\frac{k_{B}T}{6\pi \eta R_{h}}$$

where *k_B_* is Bolzmann constant, *T* is temperature, *η* is viscosity of physiological saline and *R_h_* is hydrodynamic radius of the diffusing compound. *R_h_* is further defined in Equation (3):

(3) $$R_{h}={\left(\frac{3\nu MW}{4\pi N_{A}}\right)}^{1/3}$$

where *MW* is molecular weight, *v* is the partial specific volume of protein, and *N_A_* is Avogadro’s number. Overall, these equations define the diffusional time delay for drug distribution from the vitreous to the posterior chamber.

Drug elimination from the vitreous is also limited by the physical barriers of the eye (i.e., the lens and iris). Therefore, only part (S^*^) of the surface area (*S*) bordering the vitreous and posterior chamber is available for drug elimination (Equation (4)).

(4) $$k_{el}=\frac{S^{*}}{S}*\frac{1}{T_{diff}}$$

Then, we can solve the value for the anterior clearance (*CL_vitreous_*_→*anterior*_) of the protein molecule (Equation (5)). *V_vit_* is the volume of the vitreous in the rabbit eye that is a reliable estimate for the volume of drug distribution in the vitreous [9].

(5) $$CL_{vitreous\to anterior}=k_{el}*V_{vit}$$

Drug clearance from the vitreous to the anterior chamber can be defined also as in Equation (6).

(6) $$CL_{vitreous\to anterior}=\frac{S^{*}}{S}*\frac{V_{vit}}{r_{vit}{}^{2}}*\frac{k_{B}T}{\pi \eta R_{h}}$$

In the aqueous humor, mixing of the drug takes place rapidly, like in a mixed tank [19]. Therefore, no time delays were needed to describe drug distribution in the aqueous humor. Volume of distribution (*V_ah_*) was assumed to be the anatomical volume of the aqueous humor, and clearance (*CL_ah_*) was equal with the rate of aqueous humor outflow rate in the rabbit.

Then, the elimination rate (*J_anterior_*) from the aqueous humor was defined as:

*J_anterior_* = *CL_ah_* × *c_ah_* (7)

where *c_ah_* is the drug concentration in the aqueous humor.

The parameter values for the Equations (1)–(5) are shown in Table 1.

We assumed that drug clearance posteriorly was 0 or 10% of the total clearance. The simulated protein concentrations were compared with the published experimental data for bevacizumab [14], ranibizumab [15], rituximab [16], conbercept [17], and aflibercept [18]. For the drug related parameters, see Table 2.

### 2.2. Sustained Intravitreal Drug Delivery

In addition to the bolus intravitreal injections, we also simulated controlled zero-order drug release to the vitreous using total doses that were 3 times higher than the doses of simple injections (Table 2). Clearance from the vitreous to the anterior chamber and from the aqueous humor via the outflow system were defined as described earlier for the injections.

### 2.3. Simulations

The simulations were performed with Stella Professional software (ISEE Systems Inc, version 1.1., Lebanon, NH, USA).

## 3. Results

### 3.1. Injections

**Figure 2:**
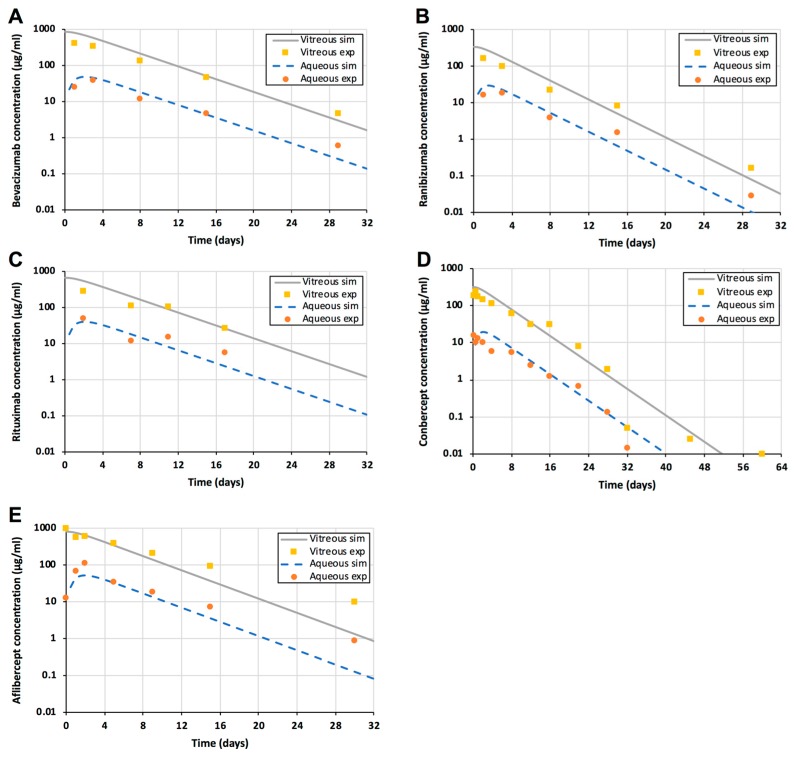
Experimental data and simulations of intravitreal injections of bevacizumab (**A**), ranibizumab (**B**), rituximab (**C**), conbercept (**D**), and aflibercept (**E**). Experimental (symbols) and simulated (lines) protein drug concentrations in the vitreous (yellow squares) and aqueous humor (orange spheres) are shown. The simulated vitreal and aqueous humor concentrations are shown as a grey solid line and a blue dashed line, respectively.

**Figure 3:**
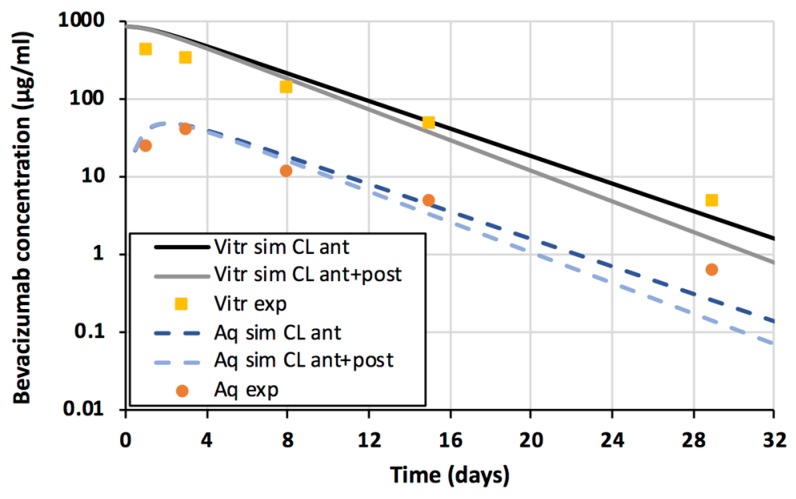
Experimental (symbols) and simulated (lines) bevacizumab concentrations in the vitreous (yellow squares) and aqueous humor (orange spheres). The simulated vitreal and aqueous humor concentrations with only anterior clearance are shown as a black solid line and a dark blue dashed line, respectively. The simulated vitreal and aqueous humor concentrations with anterior and posterior clearance are shown as a grey solid line and a light blue dashed line, respectively.

Kinetic simulations were performed with the doses that were used in the experimental publications (Table 2). The simulation model described accurately the concentration profiles of all tested biologicals in the vitreous and aqueous humor (Figure 2). The impact of additional posterior elimination was very small as shown for bevacizumab (Figure 3). Thus, vitreal diffusion (D), anatomical barrier (S*/*S*) and aqueous humor outflow were sufficient key parameters in defining the concentrations of biologicals in the vitreous and aqueous humor. Concentrations of the biologicals in the aqueous humor were about one order of magnitude lower than in the vitreous (Figure 2).

**Figure 4:**
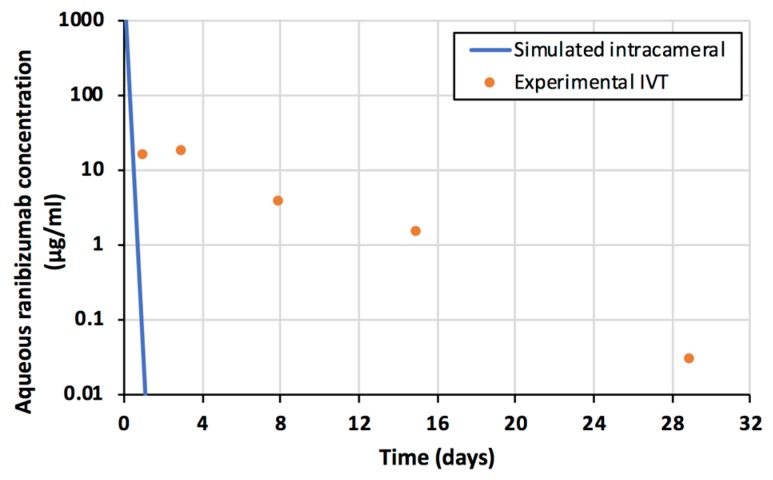
Ranibizumab concentrations in the aqueous humor after intravitreal injection (orange spheres). Simulated concentrations of ranibizumab after intracameral injection of the same dose (0.50 mg) are shown as blue line.

The declining slopes of the concentrations in the vitreous and aqueous humor are identical (Figure 2), indicating that the drug clearance from the vitreous to the anterior chamber controls the rate of drug elimination from the aqueous humor. Compared to the intracameral injection, the intravitreal injection of ranibizumab results in a more sustained drug delivery to the aqueous humor (Figure 4). Ranibizumab concentrations in the aqueous humor are similar at one month after intravitreal injection and one day after intracameral injection (Figure 4).

### 3.2. Sustained Release

**Figure 5:**
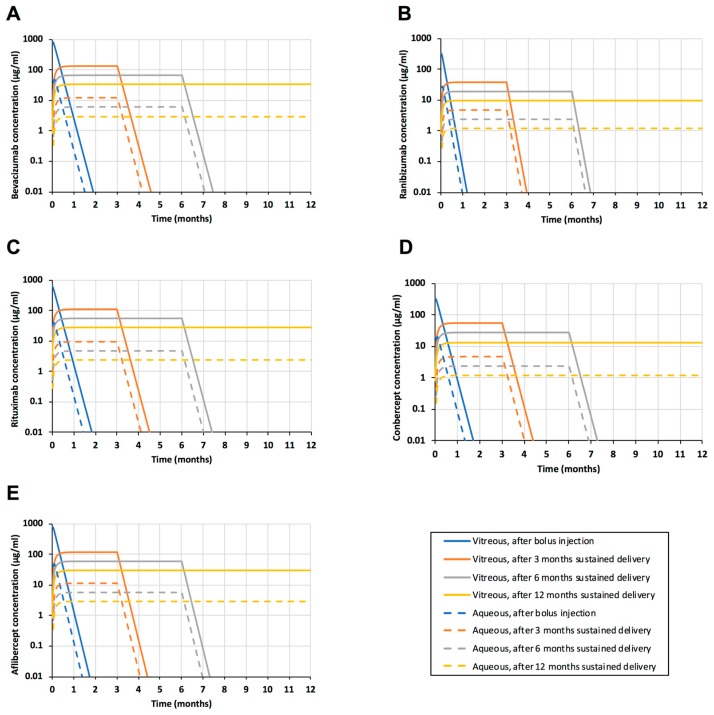
Simulations of intravitreal sustained release systems of bevacizumab (**A**), ranibizumab (**B**), rituximab (**C**), conbercept (**D**), and aflibercept (**E**). Simulated protein drug concentrations in the vitreous (solid lines) and aqueous humor (dashed lines) are shown. Simulations show intravitreal bolus injection (blue lines), and sustained delivery for 3 months (orange lines), 6 months (grey lines) and 12 months (yellow lines).

Sustained delivery of the biologicals was simulated with the model (Figure 5). Again, the role of posterior elimination was negligible (data not shown).

Bolus injections result in high peak drug concentrations in the vitreous (Figure 5). The concentrations decline at half-lives of about one week, so monthly injections are recommended for bevacizumab and ranibizumab, and two months for aflibercept. These trough concentrations of bevacizumab (at one month), ranibizumab (one month), and aflibercept (two months) can be compared with the simulated drug concentrations during sustained delivery. For bevacizumab, the release of 3.75 mg in 12 months can maintain 14 times higher concentrations than the minimum levels during the injection treatment (at 30 days) (Figure 2A). For ranibizumab, sustained delivery of 1.5 mg in 12 months results in about 160 times higher levels than the trough concentrations at 30 days after injection (Figure 2B). In the case of aflibercept, delivery of 3.60 mg in 12 months results in approximately 1.9 × 10^5^ fold higher drug levels than the trough concentrations during treatment at injection intervals of 60 days. Furthermore, the simulated controlled release dose per year was 25% and 50% of the total injected doses per year at monthly and bimonthly injection intervals, respectively. Thus, zero-order controlled drug release might lead to major dose sparing, ranging from 1 to 5 orders of magnitude, in the intravitreal anti-VEGF treatment. Overall, this means that a dose of a few milligrams per year is sufficient to maintain the concentration in the range of ≈0.1–0.2 µM (10–30 µg/mL) in the vitreous.

The levels of biologicals in the aqueous humor are approximately one order of magnitude lower than in the vitreous (Figure 5). The anterior segment drug levels of about 10–20 nM (1–3 µg/mL) can be maintained even for a year with a drug dose of a few milligrams.

## 4. Discussion

Ocular pharmacokinetics can be modeled at various levels of complexity. The most complex models utilize a finite element approach that is based on accurate anatomical 3-D representation of the eye with even >30,000 compartments [19,22]. This approach allows simulation of detailed 3-D concentration gradients within the eye, but such models can be built or used only by researchers with advanced modeling skills [19]. A previous mechanistic PK/PD model for ranibizumab [8] described the drug transfer from the vitreous to the anterior chamber in the same way as we did in this study. However, they did not report the simulated concentration profiles of ranibizumab in the aqueous humor and used fitting procedures to estimate the PK/PD parameters. Our simplified model can be used for successful bottom-up simulations as the model predicted accurately the concentrations of five biologicals in the rabbit vitreous and aqueous humor after intravitreal injections. Compared to several published models of intraocular pharmacokinetics [8,10,19,22], the model presented herein (Figure 1) is simple and easily applicable with user-friendly software that utilizes a graphical user interface. The model can be widely and conveniently used in the development of ocular delivery systems for biologicals, and is particularly useful in the design of drug payload, release rate and dosing interval of drug delivery systems.

Even though the model is useful, it has some limitations. Firstly, it is limited to the biologicals and other large molecules that do not have significant permeation across the blood ocular barriers. Secondly, the model can be used to simulate only the mean concentrations in the vitreous and aqueous humor, but not concentration gradients in the vitreous. Significant gradients do not exist in the aqueous humor [19]. Thirdly, this model neither includes pharmacodynamics nor distribution to the cells or tissues. Fourthly, the drug release rate in the model is the release rate in the vitreous, which might differ from the in vitro release rate. Reliability of the release rate depends on the quality of the in vitro release test method.

Our results support the view that intravitreal biologicals are eliminated nearly completely via the anterior route [7,9,25]. Some qualitative and outlier reports claim that biologicals are eliminated via the posterior route [26,27], but quantitative data and kinetic analyses show that anterior segment elimination of the biologicals is 4.5–50 times greater than the posterior elimination [8,19,25]. The good predictions herein (Figure 2) also support the dominant role of the anterior route, and more precisely aqueous humor outflow, in the ocular elimination of biologicals from the anterior chamber. Significant elimination through blood vessels of the anterior uvea would lead to much lower concentrations in the aqueous humor. More complex finite element models have been successful in estimating vitreal and aqueous humor kinetics of some other non-protein polymers, such as FITC-dextrans and hyaluronic acid and ranibizumab [19].

Drug diffusion in the vitreous is the rate limiting step in the elimination of biologicals via anterior route [28]. Diffusion in the vitreous can be modulated with protein size, but the size alone is not an effective means for delaying drug elimination from the vitreous, since vitreal clearance is changed only about 3-fold when molecular weight changes from 7.1 kDa to 177 kDa [25,28]. This is understandable, because diffusion in the vitreous and clearance to the anterior chamber are inversely related to the cubic root of the hydrodynamic radius (*R_h_*^1/3^) [8]. More significant delays may be reached by modulating the drug interactions with the vitreous components, such as hyaluronic acid [29]. The vitreous may become more heterogenous and its viscosity may decrease in elderly patients. Like many earlier investigators [8,19,22], we used the viscosity of saline in the simulations and reached good match between the simulations and experimental rabbit data (Figure 2). Thus, changes in the vitreous viscosity may not be a major factor in the elimination of biologicals from the vitreous.

Polymers are currently investigated as excipients in the intravitreal controlled release systems [30], but there is very little information about the elimination of the polymers from the vitreous [25]. It is known that some polymers, such as hyaluronic acid and dextran, are cleared from the vitreous solely via the anterior route [19,31]. The model presented here can also be useful in the estimation of polymer exposure of the anterior chamber tissues. However, if the polymer is degraded to very small fragments, the degradation products may be capable of crossing the blood-ocular barriers, resulting in more complicated kinetics [25]. Likewise, small molecule drugs are eliminated from the vitreous via the posterior route across the RPE and retinal capillaries [7]. Furthermore, they can be eliminated from the aqueous humor through the venous circulation of the iris and ciliary body [23,32]. Therefore, clearance from the anterior chamber may increase by an order of magnitude; for example, value of 35 µL/min was reported for pilocarpine [23]. Thus, this model should not be used as such for kinetic modeling of small molecular drugs.

Injectable and implantable long acting delivery systems are an interesting option in ocular drug delivery, particularly in the chronic treatment of age-related macular degeneration [30]. Challenges in the product development include loading capacity and stability of protein drugs in the formulation at ocular temperature for months. The simulations suggest that the controlled release may lead to significant dose sparing (per month), thereby reducing the need of the total drug dose in the formulation (a few milligrams per year). Recently, protein engineering approaches have been reported for their improved long-term stability in the vitreous [33].

Long acting intracameral implants have been developed as an alternative for the use of anti-glaucoma eyedrops that have poor patient compliance in the clinical practice [34,35]. Biologicals cannot be used as eyedrops for anterior segment treatments, because they do not permeate across the cornea and the bioavailability after sub-conjunctival delivery is less than 10% [7]. Obviously, intracameral delivery would result in 100% bioavailability in the anterior chamber, but controlled release is needed because even large molecules have aqueous humor half-lives in the range of one hour (Figure 4). Interestingly, even a bolus injection of protein drug to the vitreous should extend the concentrations in the anterior chamber to one month (Figure 2). Furthermore, the aqueous humor bioavailability of an intravitreal protein drug is almost complete, because nearly a whole dose is eliminated via the anterior route. Sampling of aqueous humor has also been used as an indicator of drug activity for intravitreally administered protein drugs, which also applies in humans [34].

Intravitreal controlled release systems can deliver biologicals to the anterior chamber, but the steady-state concentrations will be about 10 times lower than in the vitreous (Figure 2). Thus, a few milligrams of intravitreal drug in the intravitreal delivery system should be sufficient for one year if the drug is active at 10 nM concentrations. The steady-state concentrations in the aqueous humor can be estimated as the ratio of drug release rate (mass/time) and aqueous humor outflow rate (volume/time) (Equation (8)):

(8) $$C_{ss,aqueous}=\frac{C_{ss,vitreous}*CL_{vitreous\to anterior}}{aqueous\;outflow\;rate}=\frac{sustained\;release\;rate}{aqueous\;outflow\;rate}$$

The same ratio, sustained release rate/aqueous humor flow rate, also defines the steady state concentration during drug release from an intracameral implant (Equation (8)). This principle should also be applicable to the intravitreal polymers that dissolve from an implant. It should be noted that larger implants can be placed on the vitreous as compared to on the anterior chamber and the intravitreal drug administration is more widely used in clinical practice.

It is important to note, however, that the pharmacokinetics of small molecule drugs are different: anterior segment bioavailability of an intravitreal drug can be even below 10% (due to the high extent of posterior elimination) and the drug clearance from the aqueous humor is greater (even up to one order of magnitude). Thus, the steady state concentrations of small molecules, relative to the dose, can be two orders of magnitude lower than those of biologicals.

## 5. Conclusions

A simple kinetic model was successfully used to simulate the concentrations of biologicals in the vitreous and aqueous humor after intravitreal injections. The model is a useful tool in the design of administration routes and release rates of ocular biologicals.

## Figures and Tables

**Table 1:** Physical and anatomical parameter values in the simulations.

Parameter	Value	Reference
Aqueous outflow rate (mL/day)	3.49 ^a^	[20,21]
*V_vit_* (mL)	1.50	[22]
*r_vit_* (cm)	0.71	[8]
*V_aq_* (mL)	0.30	[23]
S*/*S*	0.23	[8]
*ν* (mL/g)^2^	0.73	[24]
*η* (kg/s/m)^3^	7.53 × 10^−4^	general knowledge
*k_B_* (J mol^−1^ K^−1^)	8.314	general knowledge
T (K)	298	Temperature
N_A_	6.023 × 10^23^	general knowledge

^a^ average of 2.65 [20] and 4.32 [21].

**Table 2:** Drug-related parameters.

Drug	Bevacizumab	Ranibizumab	Rituximab	Conbercept	Aflibercept
Molecular weight (kDa)	149	48	145	143	115
Bolus dose (mg)	1.25	0.50	1.00	0.50	1.20
Sustained release dose (mg)	3.75	1.50	3.00	1.50	3.60
Reference	[14]	[15]	[16]	[17]	[18]
